# Coexistence of Kabuki Syndrome and Autoimmune Thyroiditis

**DOI:** 10.4274/jcrpe.2686

**Published:** 2016-03-01

**Authors:** Fatih Gürbüz, Özge Özalp Yüreğir, Serdar Ceylaner, Ali Kemal Topaloğlu, Bilgin Yüksel

**Affiliations:** 1 Ankara Pediatric Hematology and Oncology Training and Research Hospital, Clinic of Pediatric Endocrinology, Ankara, Turkey; 2 Adana Numune Training and Research Hospital, Clinic of Medical Genetics, Adana, Turkey; 3 Intergen Genetics Center, Clinic of Medical Genetics, Ankara, Turkey; 4 Çukurova University Faculty of Medicine, Department of Pediatric Endocrinology, Adana, Turkey

**Keywords:** Kabuki syndrome, MLL2 gene, autoimmune thyroiditis, vitiligo

## DEAR EDITOR

Kabuki syndrome (KS) is a multiple congenital anomalies/intellectual disability syndrome characterized by developmental delay, specific facial features, skeletal and visceral abnormalities. This syndrome is caused by mutations in MLL2 and KDM6A gene. Autoimmune abnormalities such as idiopathic thrombocytopenic purpura, hemolytic anemia, thyroiditis, and vitiligo have been described very rarely in patients with KS (1,2,3). Herein, we present a very rare condition, KS in association with autoimmune thyroiditis and vitiligo due to a de novo heterozygous p.R2471* mutation in MLL2 gene.

**Case report:** A female patient aged 7 7/12 years presented with short stature. There was no consanguinity between her parents. She was born as a term neonate weighing 2100 g as the product of a twin pregnancy and had no perinatal complications. On physical examination, the patient was noted to have large and low-set ears, broad and arched eyebrows, elongated palpebral fissures with eversion of the lateral third of the lower eyelid, as well as high and narrow palate. She also had other phenotypic malformations such as numerous vitiligo lesions of different sizes in the neck, brachydactyly, prominent fetal finger pads, and hyperlaxity in her joints. Her height was 116.3 cm (-1.85 standard deviation score [SDS]) and weight was 21.7 kg (-0.78 SDS). She had sensorineural hearing loss (left 65 dB, right 55 dB) and moderate mental retardation (Stanford-Binet intelligence scale total score was 43). She had a normal female karyotype (46,XX). A clinical diagnosis of KS was considered.

Additionally, laboratory findings revealed autoimmune thyroiditis [thyroid-stimulating hormone (TSH): 242 mIU/L (reference range: 0.55-6.7) and free thyroxine (fT_4_) was 0.42 ng/dL (reference range: 0.91-1.92), Anti-microsomal antibody: 450.6 U/mL (reference range: 0-9), anti-thyroglobulin antibody: 2766 U/mL (reference range: 0-4)]. Thyroid ultrasonography demonstrated a rough pattern, consistent with thyroiditis. Levothyroxine (50 µg/day) replacement therapy induced a euthyroid state (TSH: 3.65 mIU/L and fT_4_: 1.15 ng/dL).

The main causes of KS are point mutations with large intragenic deletions and duplications of the histone methyl transferase MLL2 gene (1,4,5). We detected a de novo heterozygous p.R2471* (c.7411C>T) mutation in this patient. No mutations were detected in MLL2 gene in her parents and her brother. This mutation causes an early stop codon and a truncated protein. It severely affects the protein structure. To our knowledge, this mutation was not reported in KS patients to date. As this is a truncating mutation, it is most probably a disease causing mutation.

In several patients, KS was reported to be associated with autoimmune abnormalities such as idiopathic thrombocytopenic purpura, hemolytic anemia, thyroiditis, and vitiligo (3,5). The autoimmune disorders may be manifestations of abnormal immune regulation. Ming et al (3) conclude that KS is associated with an increased incidence of autoimmune disorders. In our patient, the vitiligo lesions in the neck and thyroiditis were considered to be signs revealing an autoimmune condition. We recommend that all KS patients be investigated for possible coexistence of autoimmune disorders.

Peer-review: Internal peer-reviewed.

## References

[1] Ng SB, Bigham AW, Buckingham KJ, Hannibal MC, McMillin MJ, Gildersleeve HI, Beck AE, Tabor HK, Cooper GM, Mefford HC, Lee C, Turner EH, Smith JD, Rieder MJ, Yoshiura K, Matsumoto N, Ohta T, Niikawa N, Nickerson DA, Bamshad MJ, Shendure J (2010). Exome sequencing identifies MLL2 mutations as a cause of Kabuki syndrome. Nat Genet.

[2] Miyake N, Mizuno S, Okamoto N, Ohashi H, Shiina M, Ogata K, Tsurusaki Y, Nakashima M, Saitsu H, Niikawa N, Matsumoto N (2013). KDM6A point mutations cause Kabuki syndrome. Hum Mutat.

[3] Ming JE, Russell KL, McDonald-McGinn DM, Zackai EH (2005). Autoimmune disorders in Kabuki syndrome. Am J Med Genet A..

[4] Cappuccio G, Rossi A, Fontana P, Acampora E, Avolio V, Merla G, Zelante L, Secinaro A, Andria G, Melis D (2014). Bronchial isomerism in a Kabuki syndrome patient with a novel mutation in MLL2 gene. BMC Med Genet.

[5] Giordano P, Lassandro G, Sangerardi M, Faienza MF, Valente F, Martire B (2014). Autoimmune haematological disorders in two Italian children with Kabuki syndrome. Ital J Pediatr.

